# Supplementary material for: Bromelain Confers Protection Against the Non-Alcoholic Fatty Liver Disease in Male C57BL/6 Mice
Source: Nutrients. 2020 May 18;12(5):1458. doi: 10.3390/nu12051458 (PMC7285019; doi:10.3390/nu12051458)
Supplement: Supplementary file 1 [file nutrients-12-01458-s001.pdf]

Supplementary Material

Bromelain confers protection against the non-alcoholic fatty liver disease

Po-An Hu, Chia-Hui Chen, Bei-Chia Guo, Yu Ru Kou, Tzong-Shyuan Lee*

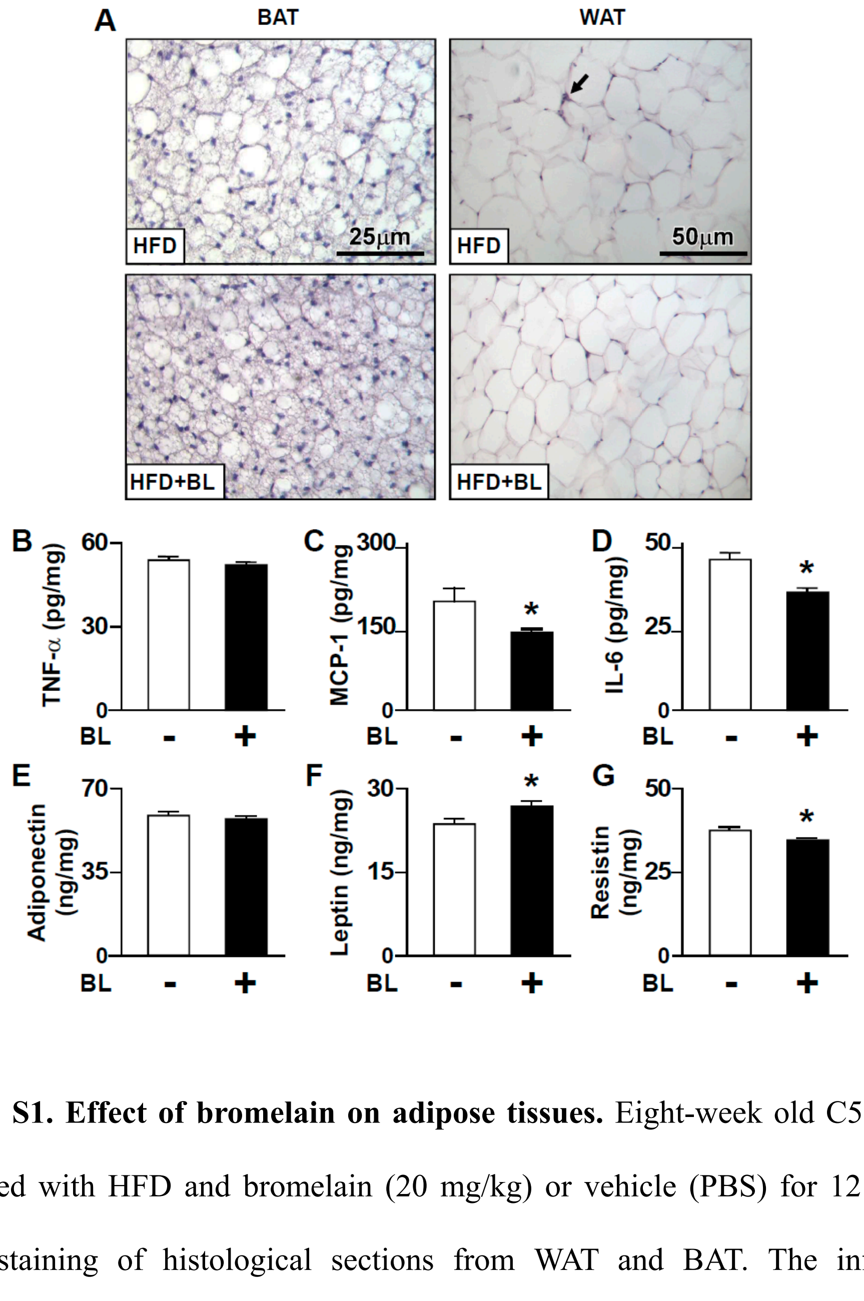

Figure S1. Effect of bromelain on adipose tissues. Eight-week old C57BL/6 mice were fed with HFD and bromelain (20 mg/kg) or vehicle (PBS) for 12 weeks. (A) H&E staining of histological sections from WAT and BAT. The infiltration of leukocytes was denoted by the arrow. (B-G) ELISA assays of TNF-α, MCP-1, IL-6, adiponectin, leptin and resistin in WAT. Results are presented by the mean ± SEM from 10 mice. * P <0.05 vs. vehicle group.

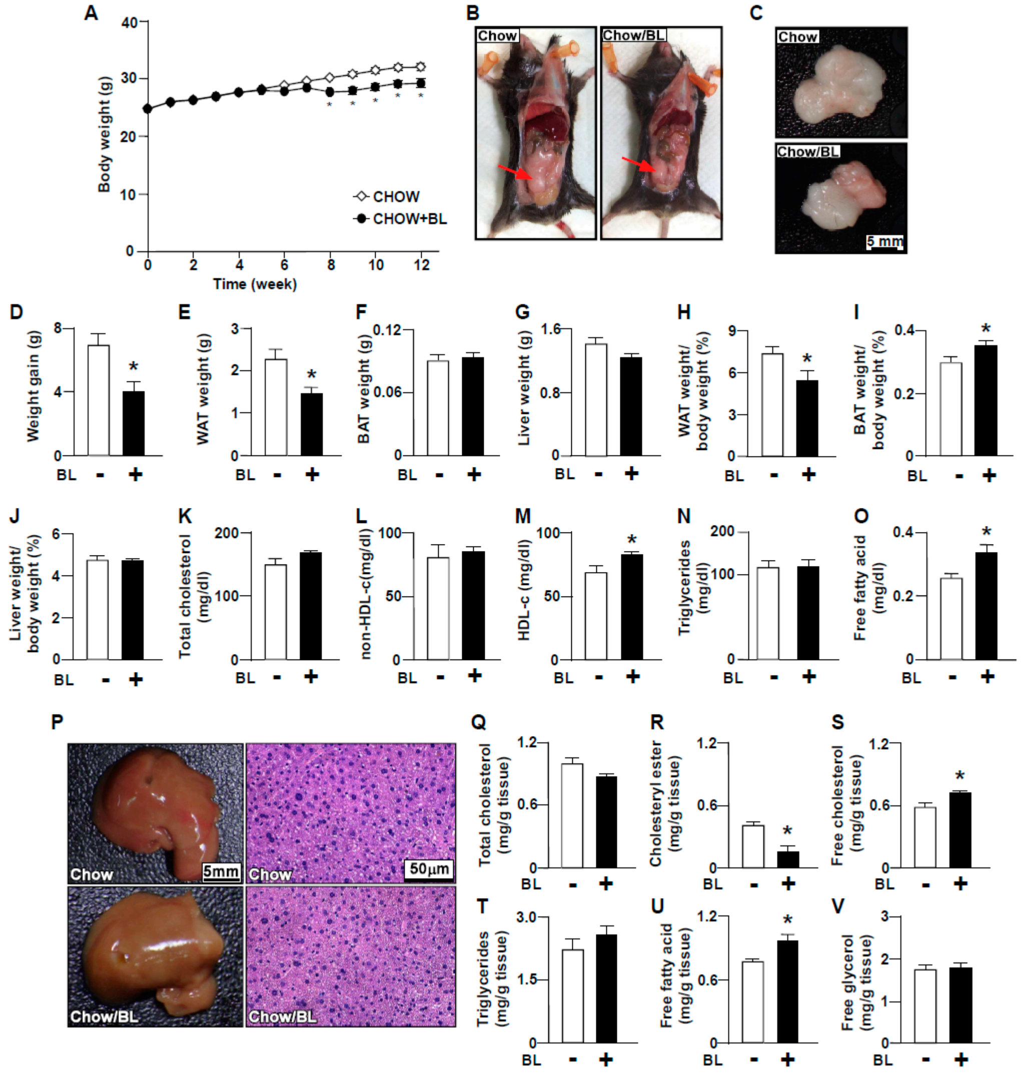

Figure S2. **Effects of bromelain on body weight, tissue weight, and blood lipids of chow diet-fed mice**. With chow-diet, eight-week old C57BL/6 mice were daily treated with bromelain (20 mg/kg) or vehicle (PBS) for 12 weeks. (A) The time-dependent change in body weight. (B and C) The images of body appearance and white adipose tissue (WAT). In (B), WAT as indicated by arrows. (D) The weight gain after chow diet treatment with or without bromelain. (E-G) The organ weights of WAT, BAT and the liver. (H-J) The ratios of organ weight to body weight in WAT, BAT and the liver. (K-O) Serum levels of total cholesterol, non-high-density lipoprotein cholesterol (non-HDL-c), HDL cholesterol (HDL-c), triglycerides, and

free fatty acids. (P) The liver appearance and representative histological images by H&E staining of the liver tissues. (Q-V) The hepatic levels of total cholesterol, free cholesterol, cholesteryl ester, triglycerides, free fatty acids, and glycerol. Results are presented by the mean ± SEM from 10 mice. * $P < 0.05$ vs. vehicle group. BL: bromelain.
